# Sun Exposure, Sun-Related Symptoms, and Sun Protection Practices in an African Informal Traditional Medicines Market

**DOI:** 10.3390/ijerph14101142

**Published:** 2017-09-28

**Authors:** Caradee Y. Wright, Tarylee Reddy, Angela Mathee, Renée A. Street

**Affiliations:** 1 Environment and Health Research Unit, South African Medical Research Council, 1 Soutpansberg Road, Pretoria 0001, South Africa; 2 Department of Geography, Geoinformatics and Meteorology, University of Pretoria, Pretoria 0002, South Africa; 3 Biostatistics Unit, South African Medical Research Council, Durban 70380, South Africa; Tarylee.Reddy@mrc.ac.za; 4 Environment and Health Research Unit, South African Medical Research Council, Houghton 2041, South Africa; Angie.Mathee@mrc.ac.za; 5 Environmental Health Department, University of Johannesburg, Johannesburg 2094, South Africa; 6 School of Public Health, University of the Witwatersrand, Johannesburg 2000, South Africa; 7 Environment and Health Research Unit, South African Medical Research Council, Durban 4091, South Africa; Renee.Street@mrc.ac.za; 8 Discipline of Occupational and Environmental Health, University of KwaZulu-Natal, KwaZulu-Natal 4041, South Africa

**Keywords:** personal sun exposure, South Africa, environmental health, informal occupational workplace, traditional health practitioners

## Abstract

Informal workers in African market trade have little formal protection against sun exposure. We aimed to examine sun exposure, sun-related symptoms, and sun protection practices in an informal occupational setting. Trained fieldworkers asked 236 workers in the Warwick Junction market about their workplace, skin and eye sensitivity and skin colour, symptoms faced at work during the summer due to heat, and preventive measures. Data were analyzed using univariate logistic regression to assess the effect of gender and the risk of experiencing symptoms to sun exposure in relation to pre-existing diseases and perception of sun exposure as a hazard. Of the 236 participants, 234 were Black African and 141 (59.7%) were female. Portable shade was the most commonly used form of sun protection (69.9%). Glare from the sun (59.7%) and excessive sweating (57.6%) were commonly reported sun-related health symptoms. The use of protective clothing was more prevalent among those who perceived sun exposure as a hazard (*p* = 0.003). In an informal occupational setting, sun exposure was high. Protective clothing and portable shade to eliminate heat and bright light were self-implemented. Action by local authorities to protect informal workers should consider sun exposure to support workers in their efforts to cope in hot weather.

## 1. Introduction

Despite their important role in the urban economy, informal workers involved in market trade have little protection against the health and safety risks they face in their workplace [1,2]. Informal workplaces, such as traditional medicine trade in an open street market, in developing countries are seldom regulated by, or inspected for, compliance to laws for formal workplace workers’ protection. Informal country-level workplace protection regulations are usually difficult to develop due to the specific social context of each workplace [3]. In South Africa, traditional medicine street markets are typically large and activities include trade in traditional medicines (plants, animals, and salts), consultations and the issue of prescriptions and health-related advice, and the preparation of traditional medicines, as well as beading, needlework, and art, among others.

Sun exposure, including both solar ultraviolet radiation (UVR) exposure and exposure to high temperatures (heat), are key health risks in an open market setting. In a changing climate, average global temperatures are expected to increase, and parts of Africa, including South Africa, are likely to see an increase double that of the global increase predicted [4]. This is likely to result in longer summers and an increase in the number of ‘very hot days’ in South Africa [5]. During extreme heat events, sun- and heat-related symptoms and outcomes experienced by informal workers in open markets may be considerable. To our knowledge, no studies have considered the sun exposure, sun-related symptoms, and sun protection practices in an African informal occupational setting. 

As part of a larger project that aimed to identify the environmental and occupational hazards of informal workers involved in the traditional medicine trade in an open street market, questions on sun and heat exposure were included in the survey questionnaire to informal workers. These results identify trends and risk factors that will help inform sun protection practices and behaviours as well as extreme heat event preparedness and coping mechanisms in the informal occupational sector of open street markets.

## 2. Materials and Methods

### 2.1. Study Site

The Warwick Junction complex is one of the largest urban traditional medicine markets in South Africa. Located in Durban in KwaZulu-Natal Province, the traditional medicine market is made up of traditional medicine suppliers, traders, and traditional health practitioners. Within the traditional medicine market, there are approximately 1000 informal traders with around 2000 assistants [6].

### 2.2. Procedures

A survey questionnaire was administered by trained fieldworkers using Mobenzi software (Mobenzi, South Africa) [7] in a structured, face-to-face interview. Eligibility criteria for study participation included being over 18 years of age, working in the traditional medicine sector, and working in Warwick Junction. Written informed consent was obtained from each participant, and data were anonymized using a unique participant identity number. Research ethics clearance was granted by the South African Medical Research Council Ethics Committee (Number EC024-8/2016, 29 August 2016). All procedures performed in studies involving participants were in accordance with the ethical standards of the South African Medical Research Council, the South African National Department of Health Ethics in Health Research and with the Helsinki Declaration and its later amendments.

### 2.3. Questionnaire

The questionnaire comprised eight sections: interview details; demography and socio-economic status; information about children; smoking history and work environment; type of work; type of products traded/prepared and related health issues; storage of products and pest control; and occupational exposure, general health, and sun exposure. Ten questions pertained to sun exposure, sun-related symptoms and sun protection practices (see Table 2, Column 1). Questions 1, 2, and 3 were about the workplace and hazards; Questions 4, 5, 6, and 7 asked about skin and eye sensitivity and skin colour; Question 8 was about symptoms faced at work during the summer/hotter months due to heat; and Questions 9 and 10 focused on preventive measures to protect against sun and heat.

### 2.4. Statistical Analyses

Data were analyzed using STATA version 14.0 (StataCorp, College Station, TX, USA) [8]. Demographic characteristics were reported as frequencies and percentages. The chi-square test or Fisher’s exact test, where appropriate, was used to test the relationship between categorical variables. Odds ratios, computed from univariate logistic regression, were used to assess the effect of gender on the risk of each health condition, respectively. Similar analyses were performed to assess the effect of pre-existing health conditions on the risk of experiencing symptoms to sun exposure.

## 3. Results

### 3.1. Sample Description Including Self-Reported Health Outcomes

Of the 236 participants in the study, 234 were Black African (2 stated ‘Other’ as their population group), 95 were male (40.3%) and 141 (59.7%) were female. South African-born participants comprised 231 individuals. Regarding age, most participants were in their 30s (32.6%). Twenty-one participants were in their 60s and 6 were in their 70s (*n* = 8 missing).

Of the self-reported, doctor-diagnosed health outcomes (Table 1), there were no reported cases of skin cancer. Hypertension (8.5%) was reported most, and there were about half as many cases of cataracts (*n* = 9). There were no statistically significant differences in self-reported doctor-diagnosed health outcomes by gender (given the small numbers) except for those who reported that they had none of the stated health outcomes, which indicated that females had a significantly higher risk of experiencing any doctor diagnosed health outcomes, compared to men.

### 3.2. Sun Exposure, Sun-Related Symptoms, and Sun Protection Practices

Of the 104 with their workplace in full sun, 68 made use of portable shade devices such as an umbrella, gazebo, or awning (Table 2), and among the 151 workers mostly sitting during the day, 55 were sitting at trade stalls that were in full sun conditions. While shade was the most reported form of sun protection used by the informal workers, there was no difference between males and females for the use of shade. In fact, there were no statistically significant differences between men and women for any of the variables in Questions 1–10, except for reported use of traditional clay as a sunscreen where all respondents who reported that they use clay were women (*p* < 0.001). 

Of those with cataracts, eight people said that the light or glare from the sun bothered their eyes sometimes and one did not. Nearly 80% reported that their skin changed when spending a lot of time in the sun, 73.7% reported that their skin went darker, and 10.1% said that their skin was sore/sensitive. Three respondents replied to ‘Other’, qualifying their response with the following: ‘itchy’, ‘itchy and rash’, and ‘I was red’. For the response ‘Other’ in reply to the question: While you are working at Warwick, do you ever use any of the following for sun protection, two respondents stated that they use calamine lotion and one mentioned a type of foundation powder. Two respondents mentioned ‘other’ symptoms that they experience during hot weather as ‘skin pinch’ (possibly tightness) and ‘thirst for water’ (thirsty).

### 3.3. Sun Exposure Symptoms and Pre-Existing Diseases

There were no statistically significant relationships between experience of sun exposure-related symptoms and self-reported doctor-diagnosed pre-existing diseases among the study participants (Table 3).

### 3.4. Sun Exposure Perceived as a Hazard and Use of Sun Protection

The use of protective clothing, such as long-sleeve shirts and long pants or long skirts, was more prevalent among those participants who reported that sun exposure is a hazard (Table 4) compared to those participants who did not report that sun exposure is a hazard.

## 4. Discussion

While skin cancer, known to be associated with excess sun exposure, is uncommon among people with dark skin [9]. Black African outdoor workers in informal trade reported three other health symptoms and impacts related to excess sun exposure, namely glare from the sun, excessive sweating, and cataracts. Solar UVR is a risk factor for cortical cataract [10]. Cataract tends to begin earlier in African populations than in comparable populations in the USA or India [11]. Any dependence of damage on skin or eye colour is unknown and, while some UV-induced eye disorders are more common in Black than in White populations, the relationship to biological differences rather than to lifestyle differences remains undefined.

Many participants noted that their skin changed when they spent significant time outdoors. Nonetheless, the use of sunscreen was low (*n* = 2; 0.9%). It may be that sunscreen is too expensive or that sunscreen is not culturally and socially acceptable among people with dark skin, as was seen in a recent study among mothers and their children [12]. Therefore, in an effort to protect themselves, several workers provided their own portable shade and wore clothing that protected their arms and legs. This is a promising finding given that, in the formal setting, the uptake of sun protection by outdoor workers is often poor and affected by the workplace, employer, and personal factors [13]. Despite the lack of a sun and heat protection policy in the Warwick Junction open market, workers did their best to protect themselves. Traditional white clay was also used by several women, although mainly applied for traditional purposes and not purposeful sun protection. Despite the reason for application, such clay is known to provide a low sun protection factor with broad-spectrum protection against harmful solar UV radiation [14].

The findings of our study may not be representative of the sun-related experiences of informal workers in other African market settings within South Africa or on the continent as a whole. Our sample was also relatively small and additional work among more participants may be useful for developing recommendations for those overseeing informal workplaces. The vastness of the informal sector is well-known [2], and our data presents a first snapshot at documenting sun-related exposures and protection among traders in an informal open market to help improve conditions and to help formulate policies and programmes to promote and ensure decent work conditions. Through our study of sun exposure, we also helped to raise awareness among informal workers of the need to protect themselves against heat and sunlight. This might be important in the future when temperatures increase and workers need to lobby local authorities to address the provision of infrastructure such as water during hot weather [15].

## 5. Conclusions

In conclusion, the present study was among the first in Africa to assess the impact of heat and sunlight on Black African informal workers in an open market setting. Overall, we did not observe meaningful associations except for wearing of protective clothing by those participants who perceived sun exposure as a hazard. This suggests that informal workers do acknowledge the need to protect themselves against excess sun exposure. Local authorities overseeing informal trade settings should consider this in the action they take, such as the provision of temporary shade structures, to ensure a safe workplace for informal workers. Awareness campaigns on heat stress in the workplace should highlight the importance of structural shade, the value of long-sleeved shirts and trousers, hats and sunscreen, and the consumption of water.

## Figures and Tables

**Table 1:** Responses to the question, “Have you ever been told by a healthcare professional that you have any of the following conditions?” for all participants and by gender.

	All (*n* = 236) *n* (%)	Male (*n* = 95) *n* (%)	Female (*n* = 141) *n* (%)	Gender OR # (95% CI)	Overall Gender Difference *p*-Value
Asthma	16 (6.8)	7 (7.4)	9 (6.4)	0.86 (0.31–2.39)	0.768
Hypertension (high BP)	20 (8.5)	5 (5.3)	15 (10.6)	2.14 (0.75–6.11)	0.146
Diabetes mellitus	12 (5.1)	3 (3.2)	9 (6.4)	2.09 (0.55–7.93)	0.269
High cholesterol	7 (3.0)	1 (1.1)	6 (4.3)	4.18 (0.49–35.27)	0.155
Obesity	6 (2.5)	2 (2.1)	4 (2.8)	1.36 (0.24–7.56)	0.726
Heart disease	5 (2.1)	1 (1.1)	4 (2.8)	2.74 (0.3–24.94)	0.351
Skin cancer	0 (0.0)	0 (0.0)	0 (0.0)	-	-
Lung cancer	0 (0.0)	0 (0.0)	0 (0.0)	-	-
Other cancer	0 (0.0)	0 (0.0)	0 (0.0)	-	-
Tuberculosis	13 (5.5)	5 (5.3)	8 (5.7)	1.08 (0.34–3.41)	0.892
Cataracts	9 (3.8)	0 (0.0)	9 (6.4)	-	-
None of the above	166 (70.3)	76 (80.0)	90 (63.8)	0.44 (0.24–0.81)	0.008

Note: # Reference group consisted of males.

**Table 2:** Questions and responses to questions on sun exposure, sun-related symptoms, and sun protection practices.

Question and Responses	All (*n* = 236) *n* (%)	Male (*n* = 95) *n* (%)	Female (*n* = 141) *n* (%)
1. Can you describe your workplace/trading area? *			
Full sun	104 (44.0)	50 (52.6)	54 (38.2)
Partly shaded	100 (42.3)	33 (34.7)	67 (47.5)
Full shade	22 (9.3)	12 (12.6)	10 (7.0)
Protection from rain	19 (8.0)	8 (8.4)	11 (7.8)
Windy	97 (41.1)	38 (40.0)	59 (41.8)
Closed room	11 (4.6)	9 (9.4)	2 (1.4)
Other ^@^	44 (18.6)	16 (16.8)	28 (19.8)
2. In your working day, are you mostly:			
Sitting	151 (64.0)	50 (52.6)	101 (71.6)
Standing	54(22.9)	35 (36.8)	19 (13.4)
Walking about	14 (5.9)	5 (5.2)	9 (6.3)
Other ^@^	17 (7.2)	5 (5.2)	12 (8.5)
3. Is sun exposure a hazard that you are exposed to during your work? *			
Yes	134 (56.7)	54 (56.8)	80 (56.7)
4. If you spend a lot of time in the sun does your skin change?			
Yes	187 (79.2)	73 (76.8)	114 (80.8)
No	49 (20.8)	22 (23.1)	27 (19.1)
5. Does your skin change? *			
Darker	174 (73.7)	71 (74.7)	103 (73.0)
Sore/sensitive	24 (10.1)	7 (7.3)	17 (12.0)
Other	3 (1.2)	0 (0.0)	3 (2.1)
6. Does light or glare from the sun bother your eyes sometimes?			
Yes	141 (59.7)	54 (56.8)	87 (61.7)
No	95 (40.3)	41 (43.1)	54 (38.2)
7. Is the skin colour on the inside of your upper arm:			
Black	19 (8.1)	9 (9.4)	10 (7.0)
Dark brown	7 (3.0)	4 (4.2)	3 (2.1)
Brown	50 (21.2)	22 (23.1)	28 (19.8)
Light brown	116 (49.2)	46 (48.4)	70 (49.6)
White	44 (18.6)	14 (14.7)	30 (21.2)
8. What symptoms do you face at work during the summer/hotter months due to heat? *			
Excessive sweating	136 (57.6)	61 (64.2)	75 (53.1)
Muscle cramps	44 (18.6)	12 (12.6)	32 (22.6)
Tiredness/weakness	62 (26.2)	19 (20.0)	43 (30.4)
Dizziness	44 (18.6)	18 (18.94)	26 (18.4)
Headache	53 (22.4)	17 (17.8)	36 (25.5)
Nausea/vomiting	20 (8.4)	6 (6.3)	14 (9.9)
Fainting	1 (0.4)	0 (0.0)	1 (0.7)
No symptoms experienced	46 (19.4)	18 (18.9)	28 (19.8)
Other	2 (0.8)	1 (1.0)	1 (0.7)
9. While you were working at Warwick, do you ever use any of the following for sun protection? *			
Hat	89 (37.7)	45 (47.3)	44 (31.2)
Sunglasses	4 (1.6)	2 (2.1)	2 (1.4)
Shade (umbrella, gazebo, awning, tree, trading stall)	165 (69.9)	72 (75.7)	93 (65.9)
Sunscreen	2 (0.8)	2 (2.1)	0 (0.0)
Long-sleeve clothing with the purpose of blocking the sun from your skin	70 (29.6)	24 (25.6)	46 (32.6)
Traditional clay (relate to recent paper on clays)	73 (30.9)	0 (0.0)	73 (51.6)
Other	8 (3.3)	1 (1.0)	7 (4.9)
10. Do you drink more liquids during the summer months?			
Yes	236 (100.0)	95 (100.0)	141 (100.0)
No	0 (0.0)	0 (0.0)	0 (0.0)

* Multiple responses were possible where participants could select all responses that applied to them; ^@^ Reported elsewhere as considered not relevant for sun exposure.

**Table 3:** The relationship between the experience of sun exposure-related symptoms and pre-existing diseases among the study participants. None of the odds ratios were statistically significant.

Sun Exposure-Related Symptom	Total *n* (%)	No Pre-Existing Disease *n* (%)	Any Pre-Existing Disease *n* (%)	Odds Ratio (95% Cl)	*p*-Value
Excessive sweating	136 (57.6)	98 (59.4)	38 (53.5)	0.79 (0.45–1.38)	0.403
Muscle cramps	44 (18.6)	26 (15.8)	18 (25.4)	1.82 (0.92–3.58)	0.085
Tiredness/weakness	62 (26.3)	39 (23.64)	23 (32.39)	1.55 (0.84–2.86)	0.162
Dizziness	44 (18.6)	27 (16.4)	17 (23.9)	1.61 (0.81–3.19)	0.173
Headache	53 (22.5)	34 (20.6)	19 (26.8)	1.41 (0.74–2.69)	0.3
Nausea	20 (8.5)	14 (8.5)	6 (8.5)	1.00 (0.37–2.71)	0.993
Fainting	1 (0.4)	0 (0.0)	1 (1.41)	-	0.301
No symptoms	46 (19.5)	37 (22.4)	9 (12.7)	0.50 (0.23–1.11)	0.087
Thirsty	1 (50.0)	1 (100.0)	0 (0)	1	-
Other symptoms	2 (0.8)	1 (0.61)	1 (1.41)	2.34 (0.14–37.99)	0.549

**Table 4:** The relationship between whether a participant agreed that sun exposure is a hazard and the use of sun-protective equipment/items.

Sun Protection Equipment/Item	Total (*n* = 236) (%)	Sun Exposure Is Not Hazard (*n* = 102) (%)	Sun Exposure Is a Hazard (*n* = 134) (%)	*p*-Value
Hat	89 (37.7)	32 (31.4)	57 (42.5)	0.08
Sunglasses	4 (1.7)	2 (2.00)	2 (1.5)	0.782
Shade (umbrella, gazebo, awning, tree, trading stall)	166 (70.3)	73 (71.6)	93 (69.4)	0.718
Sunscreen	2 (0.9)	0.00	2 (1.5)	0.215
Protective clothing (long-sleeves shirts, long pants)	70 (29.7)	20 (19.6)	50 (37.3)	0.003
Traditional clay	73 (30.9)	25 (24.5)	48 (35.8)	0.063
Other	8 (3.4)	3 (2.9)	5 (3.73)	0.74
